# Positive Correlation between Lipin-1 and Lipin-2 Expressions and Hepatic T1 Values in IUGR Rats

**DOI:** 10.2174/0115734056296733250514081236

**Published:** 2025-05-26

**Authors:** Tao Wang, MingZhu Deng, Alpha Kalonda Mutamba, XiaoRi He, Jing Bian, DuJun Bian

**Affiliations:** 1 Department of Pediatric, The Second Xiangya Hospital of Central South University, Changsha, China; 2 Department of Clinical Medicine, School of Medicine, Hunan Normal University, Changsha, China; 3 Department of Radiology, The Second Xiangya Hospital of Central South University, Changsha, China

**Keywords:** Intrauterine growth restriction, T1 mapping, Hepatic T1 values, Lipin-1, Lipin-2, Visceral adipose tissues

## Abstract

**Background:**

Intrauterine growth restriction (IUGR) is associated with long-term metabolic disturbances, including obesity. Changes in hepatic lipid metabolism and adipose tissue function, mediated by lipin-1 and lipin-2, may contribute to these outcomes.

**Aim:**

This study aimed to investigate the correlation between lipin-1 in visceral adipose tissues (VATs) and lipin-2 in the liver. It also examined hepatic T1 values using T1 mapping in IUGR rats.

**Objective:**

The objective of this study was to explore the metabolic mechanisms linking IUGR and adult obesity by analyzing molecular and imaging markers.

**Methods:**

Pregnant rats were fed either a low-protein diet (10%) to induce IUGR or a normal-protein diet (21%) as a control. Male offspring underwent conventional magnetic resonance imaging and native T1 mapping using a 3.0 T whole-body MR scanner at days 21, 56, and 84 post-birth. Liver tissues and VATs were collected for analysis. Lipin-1 and lipin-2 expression levels were measured using Western blot and real-time quantitative PCR.

**Results:**

The IUGR group exhibited significantly higher mRNA and protein expression levels of lipin-1 and lipin-2 compared to the control group at days 21, 56, and 84 after birth. Additionally, the IUGR group demonstrated significantly higher hepatic T1 values than the control group at the corresponding time points. Positive correlations were observed between the protein and mRNA expression levels of lipin-1 and hepatic T1 values. Similarly, the protein and mRNA expression levels of lipin-2 were positively correlated with hepatic T1 values. All results were statistically significant (P<0.05).

**Conclusion:**

The upregulation of lipin-1 and lipin-2 expressions was found to be linked to elevated hepatic T1 values, potentially contributing to adult obesity in IUGR rats.

## 1 INTRODUCTION

During fetal development, environmental stimuli, such as malnutrition, can significantly affect fetal growth and development, posing risks for complications across generations [1-3]. Intrauterine growth restriction (IUGR) is one such phenomenon that causes alterations in organogenesis, tissue development, and metabolism, predisposing offspring to obesity and metabolic disorders later in life [4-6]. Maternal low-protein diets are a well-established cause of IUGR, leading to catch-up growth and the development of obesity in adulthood [7-9]. Despite strong evidence linking IUGR to an increased risk of metabolic diseases, the underlying mechanisms remain poorly understood [10].

IUGR is closely associated with altered liver development in humans and animal models, often resulting in impaired liver function and obesity. Individuals with poor in-utero growth have an elevated risk of hepatic disorders, including non-alcoholic fatty liver disease (NAFLD) [11, 12]. However, the specific mechanisms linking IUGR to NAFLD are not yet fully elucidated [13, 14].

Traditionally, liver lipid assessment relies on percutaneous liver biopsy. While effective, this invasive procedure carries risks and complications [15]. Recent advancements have introduced non-invasive methods for liver tissue characterization, with T1 mapping emerging as a promising technique [16]. T1 mapping measures the longitudinal relaxation time constant (T1), which reflects changes in water content or the local molecular environment of the liver. Altered T1 values have been identified as biomarkers for various liver pathologies, providing a quantitative and non-invasive means to assess tissue characteristics [17].

Liver and adipose tissues play pivotal roles in lipid metabolism, and structural abnormalities in these tissues can disrupt metabolic homeostasis [18, 19]. The lipin gene family, which includes lipin-1, lipin-2, and lipin-3, is integral to lipid metabolism and liver development. Lipin-1 is highly expressed in adipose tissue, while lipin-2 predominates in the liver, suggesting tissue-specific functions [20, 21]. Previous studies have focused on lipin-1 and lipin-2, while lipin-3 activity remains poorly understood and was also not assessed in this study due to limited availability [22, 23].

Notably, most research works have investigated lipin expression in the visceral adipose tissues (VATs) of male rats, leaving gaps in understanding its effects on female rats and subcutaneous adipose tissue (SAT) [24]. Furthermore, the correlation between lipin expression and hepatic lipid metabolism mechanisms remains incomplete [25].

This study aimed to investigate the correlation between lipin-1 in VATs, lipin-2 in the liver, and hepatic T1 values using T1 mapping in IUGR rats. We employed immunohistochemistry and western blot analyses to detect lipin-1 and lipin-2 protein expression across various time points. Simultaneously, T1 mapping quantified hepatic T1 values using a 3.0 T whole-body MR scanner. This investigation emphasizes the potential link between lipin gene expression and liver pathology in the context of IUGR.

## 2 MATERIALS AND METHODS

### 2.1 Animal Preparation

All animals were provided by the Animal Center of The Second Xiangya Hospital of Central South University (No. 2020169). Procedures adhered to the National Health Commission of China’s guidelines (Guide for the Care and Use of Laboratory Animals) and were approved by the Ethics Committee of the Second Xiangya Hospital.

### 2.2 Experimental Design

Twenty Sprague-Dawley rats (10 males and 10 females, weighing 250-300g; the age of pregnant Sprague–Dawley rats were 3 months old, and the ages of the control and IUGR model group were 3, 8, and 12 weeks post birth) were housed under controlled conditions with constant temperature, humidity, and a 12:12-hour light/dark cycle. Female rats were paired with males in a 1:1 ratio for mating, with the detection of vaginal plug marking day 1 of pregnancy.

Pregnant rats were randomly assigned to two groups: a control group fed a 21% protein diet, and an IUGR group fed a low-protein diet (10% protein). During the 21-day lactation period, both groups were provided with a normal protein diet (21%). IUGR defines newborns with a birth weight two standard deviations lower than the average weight for the same gestational age. Male pups from each group were fed a normal protein diet post-weaning until 84 days of age.

Growth parameters, including body weight, BMI, body length, and waist circumference, were measured on days 21, 56, and 84. Based on the three time points used in our study, the comparable sexual developmental stages between rats and humans are as follows: at days 21 after birth represents early adolescence, at days 56 after birth represents mid-adolescence or young adulthood, and at days 84 after birth signifies late adolescence or early adulthood. Waist circumference was assessed in sedated pups by measuring at the midpoint between the xiphoid and hind limbs.

### 2.3 Sample Collection

Male pups were euthanized at 21, 56, and 84 days post-birth. At the end of the experiments, euthanasia using carbon dioxide (CO_2_) was performed by placing the rats in a euthanasia chamber filled with CO_2_ gas. Liver tissues were collected at these same time points, while visceral adipose tissues were harvested at 21, 56, and 84 days post-birth. Both liver and visceral adipose tissues were promptly removed, frozen in liquid nitrogen, and stored at -80°C until processing. Ten rats from each group were included at each time point in this study.

### 2.4 Real-Time Reverse Transcription Quantitative PCR (RT-qPCR)

Lipin-1 and lipin-2 mRNA expressions were assessed using RT-PCR. Reagents were sourced from a reverse transcription system kit (Fermentas) and SYBR Green qPCR Mix (TOYOBO, Japan). For lipin-1, the forward and reverse primers used were 5'-TCACTACCCAGTACCAGGGC-3' and 5'- TGAGTCCAATCCTTTCCCAG-3', respectively. For lipin -2, primers were 5'-GAACTTCCTGAATCGCCTCT-3' and 5'-CCGGATC GGTGTCACTTT-3'. β-actin served as the internal control, with primers: 5'-AGGGGCCGGACTCGTCATACT-3' and reverse primer: 5'-GGCGGCACCACCATGTACCCT-3'. PCR data analysis was performed using the comparative ΔΔCT method.

### 2.5 Western Blot

Total protein was extracted from tissues using RIPA buffer (Auragene, Changsha, China) supplemented with protease inhibitors (Auragene, P019A). Protein concentration was determined by the Bradford method. SDS-PAGE separation (50 micrograms of protein) was followed by transfer to nitrocellulose membranes (Millipore, Bedford, MA, USA). Membranes were blocked with 3% BSA in TBST, incubated with primary antibodies overnight at 4°C, and then probed with horseradish peroxidase (HRP)-conjugated secondary antibodies. Rabbit anti-lipin-1 and anti-lipin-2 antibodies (Abcam, Cambridge, UK) and mouse anti-β-actin (Auragene, Changsha, China) were used. Densitometry analysis was performed using Image-Pro Plus software 6.0, with data normalized to β-actin.

### 2.6 MRI Procedure

MRI was conducted using a 3.0 Tesla imaging system (MAGNETOM Skyra, Siemens, Germany). Rats were anesthetized with 50 mg/kg pentobarbital sodium and positioned in a 12-channel rat coil. Fasting rats (≥6 hours) were placed in a cradle and imaged using various protocols, including T2-weighted, T1-weighted, Diffusion-Weighted Imaging (DWI), Variable Flip angle T1 mapping, T2 mapping-weighted imaging, and MR spectra. Variable Flip angle T1 mapping images were obtained in axial planes. The following parameters were used: repetition time (TR)/echo time (TE)= 15/3.87 ms, mean number of signals (MNS)=2, flip angle1 (FA)=5°, FA2=26°, bandwidth (BW)= 400 Hz/Px, matrix size= 320×320, and acquisition time= 3:09. MR quantifications were processed using MR workspace, and hepatic T1 values were calculated from ROI (region of interest) placed in the liver parenchyma by an expert radiologist. All ROIs were placed on the same slice, showing the maximum area of the liver parenchyma, ensuring they were kept away from large bile ducts, blood vessels, subcutaneous fat, and the intestine.

### 2.7 Statistical Analysis

The data were presented as mean±SD and analyzed using SPSS, version 23.0. Paired t-tests were employed for statistical analysis. To assess the correlation between lipin expressions and MR quantification of hepatic T1 values, Pearson’s correlation test was conducted. Results were deemed statistically significant at a p-value of <0.05.

## 3 RESULTS

The induction of the IUGR model involves maintaining pregnant mice on a low-protein diet throughout gestation. This results in the development of IUGR (5.23±0.54g), identified using the criteria of the average birth weight of control group pups (7.36±0.62g) minus 2 standard deviations (P<0.05). The experimental groups showed a significantly increased likelihood of the IUGR group compared to the control group (94.07%vs.3.48%, P<0.05).

At 21 days, male pups of IUGR rats exhibited significantly lower body weight, BMI, and body length compared to pups of the control rats, with no significant difference in waist circumference. However, at 56 days, pups of IUGR rats showed significantly higher body weight, BMI, and waist circumference, while body length did not show a significant difference compared to control rat pups. By 84 days, body weight, BMI, and waist circumference in pups of IUGR rats were all significantly higher than those in pups of the control rats (Table **1**).

The expression of lipin-1 mRNA and protein in the VATs of male pups from IUGR rats was significantly increased at 21 days (p=0.0001, p=0.001), 56 days (p=0.041, p=0.001), and 84 days (p=0.001, p=0.001) post-birth when compared to control rats (Table **1**). Similarly, the expression of lipin-2 mRNA and protein in liver tissues of IUGR rat pups showed significant increases at 21 days (p=0.029, p=0.027), 56 days (p=0.001, p=0.044), and 84 days (p=0.025, p=0.001) post-birth in comparison to control rats (Table **1**).

**Fig (1):**
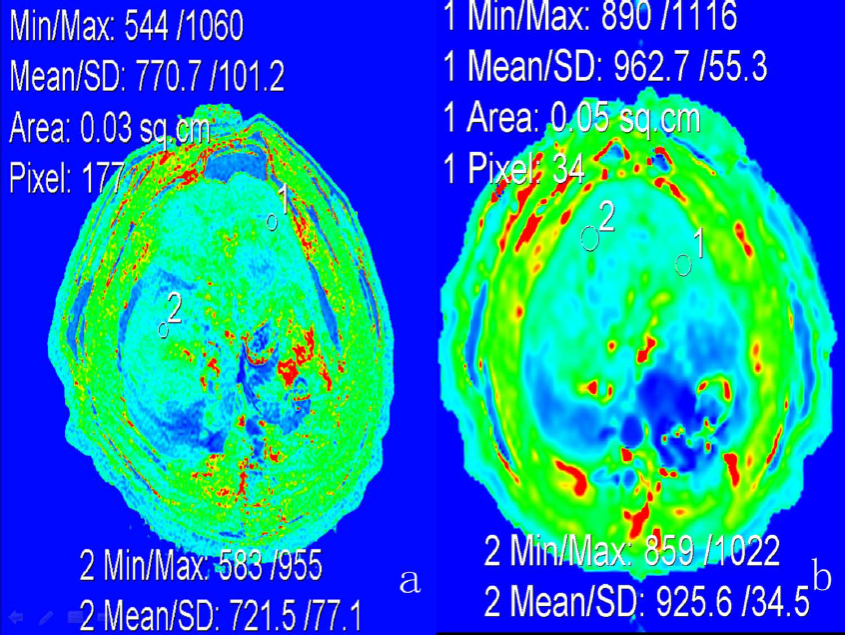
Comparison of MR T1 mapping technique quantified the hepatic T1 value between the two groups at 12 weeks. (**a**) Representative control group. (**b**) Representative IUGR group. At week 12 after birth, the IUGR group had a significantly higher hepatic T1 value than the control group (P<0.05).

At all three time points, the native T1 values were significantly higher in the livers of IUGR rats compared to sham livers. Specifically, hepatic T1 values in the livers of pups from IUGR rats were significantly elevated at 21 days (p<0.05), 56 days (p<0.05), and 84 days (p<0.05) post-birth in contrast to the livers of control rat pups (Fig. **1**).

Significant positive correlations were observed between hepatic T1 values and the expression of lipin-1 protein (r = 0.642, p<0.05) and mRNA (r = 0.553, p<0.05). Additionally, significant positive correlations were found between hepatic T1 values and the expression of lipin-2 protein (r = 0.611, p<0.05) and mRNA (r = 0.525, p<0.05).

## 4 DISCUSSION

Recent studies in both humans and animals suggest that both insufficient and excessive nutrition can influence epigenetic modifications, providing insight into the developmental origins of health and disease (DOHaD) [26-29]. Animal models of intrauterine growth restriction (IUGR) support the hypothesis that in-utero deficiencies impair growth and disrupt triglyceride metabolism, leading to obesity in adulthood, with potential intergenerational effects [30-32]. Interestingly, adipose dysregulation follows distinct mechanisms in female IUGR rats compared to males, possibly involving sex steroid-related pathways for adipose deposition in females [33-35]. This study utilized a well-established low-protein rat model to examine the molecular profiles of lipin-1 and lipin-2 in the liver and visceral adipose tissue (VAT) of male offspring from early postnatal stages to adulthood. Offspring from undernourished mothers exhibited smaller birth weights and stunted growth at 7 days, followed by catch-up growth at 21 days, surpassing normal levels in weight and crown-rump length. By 56 days, excessive adipose accumulation occurred, leading to greater adiposity and obesity by 84 days in IUGR rats.

Non-invasive imaging techniques, particularly native T1 mapping, have emerged as powerful tools for quantifying liver fat content [36]. Native T1 mapping measures T1 relaxation times in milliseconds without the need for contrast agents, reflecting signal changes in the molecular environment of water molecules in tissues [37]. This technique, increasingly used in clinical practice to assess liver steatosis and iron overload [38], has the advantage of avoiding contrast agents and offering routine imaging with high-quality results [39]. T1 mapping is a promising non-invasive method for assessing liver health in IUGR (intrauterine growth restriction) patients. By using MRI to quantify liver tissue properties, such as water content, fibrosis, and lipid accumulation, T1 mapping offers a safe and reproducible alternative to invasive liver biopsies [40]. This makes it particularly valuable for monitoring IUGR patients, who are at higher risk of developing hepatic steatosis, fibrosis, and metabolic disorders [41].

A key advantage of T1 mapping is its ability to detect subtle early changes in liver composition, such as alterations in water-to-fat ratios and extracellular matrix content [42], which may result from disrupted lipid metabolism in IUGR. Early detection enables timely interventions to reduce the risk of long-term complications like NAFLD and metabolic syndrome. Additionally, its non-invasive nature allows for repeated assessments over time, making it ideal for tracking disease progression or treatment responses in pediatric and adolescent populations, where invasive procedures are less desirable [43]. By enabling early diagnosis and personalized management strategies, T1 mapping helps bridge the gap between molecular findings and clinical practice [44]. It also facilitates broader research into the long-term hepatic and metabolic consequences of IUGR, providing a valuable tool for improving patient outcomes.

In this study, liver T1 measurements correlated significantly with lipin-1 and lipin-2 gene expressions. Unlike histological methods, T1 mapping provides high-volume coverage, requires less post-processing time, and does not necessitate animal sacrifice. Moreover, it allows repeated measurements over time, facilitating longitudinal studies and follow-up examinations. Despite challenges posed by motion artifacts due to respiration, bowel peristalsis, and vascular pulsations, advances in fast imaging sequences are expected to overcome these limitations, further enhancing the utility of T1 mapping in preclinical and clinical research [45, 46]. The adoption of MR T1 mapping in preclinical studies could reduce animal numbers and enable non-invasive, quantitative comparisons within the same subjects across time, ultimately benefiting both research and clinical practice.

The maternal nutrient restriction affects offspring gene expression through epigenetic mechanisms, leading to increased adipose tissue and heightened lipin expression [47, 48]. Offspring from malnourished dams show alterations in adipogenesis, lipogenesis, adipokine expression, thermogenesis, and low-grade inflammation. Lipin-1 and lipin-2 are critical regulators of lipid metabolism in the liver, playing key roles in triglyceride synthesis and phospholipid production [49]. Dysregulation of these proteins disrupts lipid homeostasis, contributing to hepatic steatosis, fibrosis, and metabolic disorders [50]. Overexpression of lipin-1 in adipocytes has been linked to increased triacylglycerol (TAG) accumulation [51]. Lipins, particularly lipin-1, are key enzymes in lipid synthesis [52], performing dual functions in phosphatidate phosphatase activity to help maintain lipid metabolic balance. Studies have reported that lipin-1 plays a crucial role in lipid metabolism, including triglyceride synthesis and secretion. Enhanced expression of lipin-1 in hepatocytes stimulates triglyceride synthesis, while reduced expression decreases triglyceride secretion [53]. Additionally, research indicates that lipin-1 expression is regulated by hormonal factors, such as glucocorticoids, which influence adipocyte differentiation and lipid metabolism [54]. The observed increase in lipin-1 expression in the control group after 12 weeks may be attributed to natural physiological changes associated with aging and metabolic regulation. Age-related changes in lipid metabolism and hormonal regulation could lead to an increase in lipin-1 expression over time in control animals. However, the exact mechanisms underlying this observation require further investigation. While lipin-1 has been extensively studied, the role of lipin-2, although also exhibiting phosphatidate phosphatase (PAP) activity, remains less understood [55]. As animals age, their lipid metabolism adjusts to maintain balance, and lipin-2 likely plays an increasing role in supporting these processes [56]. Hepatic T1 values, measured via MRI, reflect liver composition, particularly water and fat content. It is important to note that besides liver fat fraction, T1 values are also affected by liver iron content, inflammation, and liver stiffness [57].

The significant increase in lipin-2 expression in the control group after 12 weeks compared to earlier time points can be attributed to age-related metabolic adaptations [58]. This may also reflect a compensatory mechanism, as lipin-1 expression increases with age, and lipin-2 may take over to ensure proper lipid synthesis and storage. Additionally, hormonal and inflammatory signals, which influence lipin-2 expression, could become more prominent over time, driving its up-regulation [59]. Lastly, the role of lipin-2 in preventing liver lipid toxicity and maintaining hepatic function may become more critical as the animals mature [60]. IUGR may disrupt liver development, impairing liver function and leading to obesity.

The findings from this study have important clinical implications for the management of IUGR and metabolic diseases. The significant increase in lipin-1 and lipin-2 expressions in the visceral adipose tissues and liver of IUGR rat pups suggests a potential mechanism for the altered lipid metabolism and fat storage observed in IUGR [61]. These findings highlight the role of these molecular markers in metabolic disturbances that can persist postnatally, potentially contributing to the development of metabolic diseases, such as non-alcoholic fatty liver disease (NAFLD) and insulin resistance [62]. Clinically, these results suggest that monitoring lipin-1 and lipin-2 expression could serve as early biomarkers for identifying individuals at risk for developing metabolic disorders, particularly those with a history of IUGR. Early interventions targeting lipid metabolism, possibly through dietary modifications or pharmacological agents aimed at modulating lipin expression, could help prevent or mitigate these long-term metabolic risks.

Additionally, the positive correlations between hepatic T1 values (from imaging techniques like MRI) and the expression of lipin proteins suggest that advanced imaging could be used to non-invasively monitor liver fat content and metabolic changes in patients with IUGR or other metabolic disorders [63]. This imaging biomarker could be valuable for assessing the severity of liver fat accumulation and tracking the efficacy of interventions over time. In clinical practice, integrating these molecular and imaging markers could lead to more personalized treatment plans for IUGR patients and others at risk for metabolic diseases. Future research should focus on validating these markers in larger, more diverse populations, investigating their role in other tissues involved in metabolic regulation, and exploring potential therapeutic strategies targeting lipin expression to prevent or treat metabolic diseases.

**Fig. (2):**
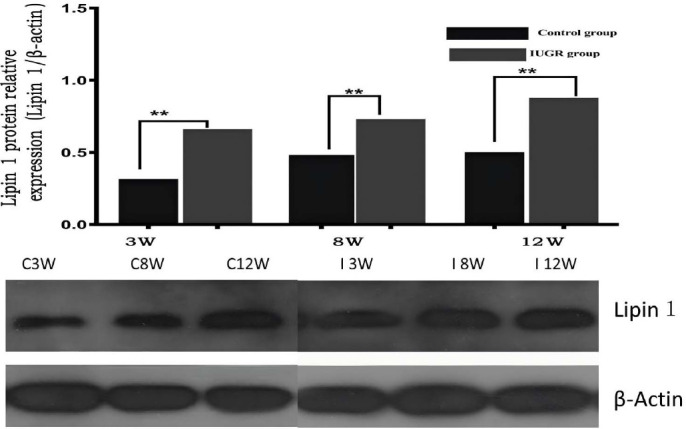
IUGR increases the expression of lipin-1 protein in visceral adipose. Quantification of IUGR male lipin-1 protein in visceral adipose relative to control (top).Western blot of lipin-1 protein expression in adipose tissues (upper band) in male offspring from control and IUGR groups. Data were normalized to β-actin (lower band) and presented as fold difference. β-actin was comparable between IUGR and control offspring at both ages.

**Fig. (3):**
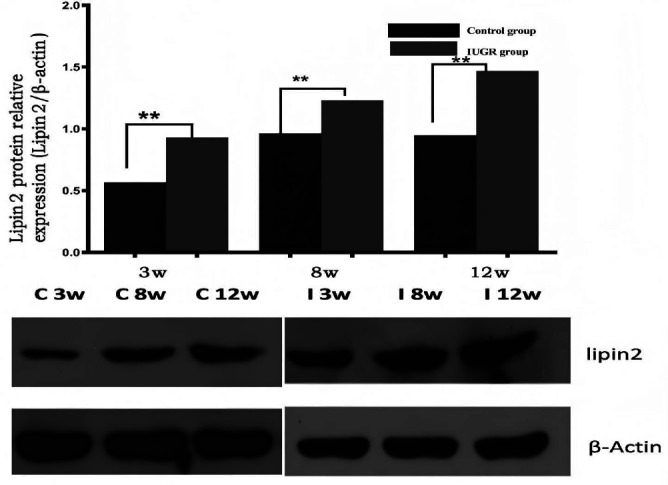
IUGR increases the expression of lipin-2 protein in liver tissues. Quantification of lipin-2 protein in liver tissues of IUGR male offspring relative to controls (top). Western blot analysis of lipin-2 protein expression in liver tissues (upper band) from male offspring of control and IUGR groups. Data were normalized to β-actin (lower band) and presented as fold differences. β-actin expression was comparable between IUGR and control offspring at both ages.

**Fig. (4):**
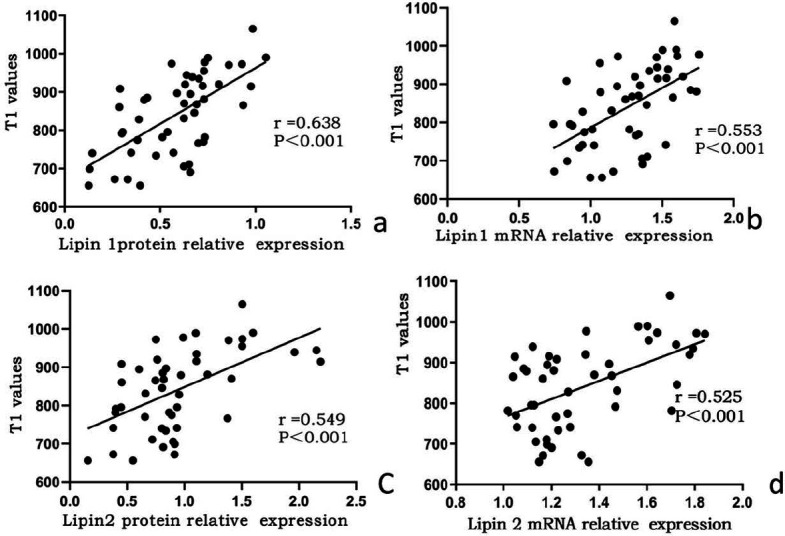
The scatter plots representing correlation between lipin protein and mRNA (x-axes) and the hepatic T1 values (y-axes). From left top (lipin-2 protein), right top (lipin-1 protein) to left bottom (lipin-2 mRNA), right bottom (lipin-1 mRNA).

The liver, a critical organ in IUGR monitoring, is susceptible to metabolic changes, such as fatty infiltration [64, 65]. In IUGR models, altered lipin-1 and lipin-2 expressions predispose individuals to metabolic disorders and liver abnormalities. In this study, we observed that elevated hepatic T1 values in IUGR rats correlated with increased expression of lipin-1 and lipin-2, as confirmed by immunohistochemistry and Western blot analysis. Elevated lipin-1 and lipin-2 expressions enhance triglyceride synthesis, leading to impaired lipid metabolism and fat accumulation, thus triggering fibrosis and inflammation, which increase extracellular water content and elevate T1 values. This study highlights the correlation between lipin-1 and lipin-2 expression and hepatic T1 values, emphasizing T1 mapping as a non-invasive biomarker for early metabolic disruptions. These findings contribute to understanding the molecular impact of IUGR and support the potential of T1 mapping for early diagnosis and management of metabolic disorders. Our findings highlight the association of higher lipin expression with increased liver T1 values in male IUGR rat offspring, both in the short and long term. Further biochemical analysis is planned to explore the correlation between the expression levels of lipin-1 and lipin-3, along with lipid biochemical analysis in plasma and adipose tissue, as well as the assessment of antioxidant and inflammatory biomarkers (Figs. **2**-**4**).

## CONCLUSION

In conclusion, this study demonstrated the efficacy of native T1 mapping as a non-invasive method for measuring total hepatic T1 values in IUGR rats, providing a strong foundation for future longitudinal clinical and research investigations. Hepatic T1 values were significantly correlated with lipin-2 expression in liver tissue and lipin-1 expression in visceral adipose tissue, highlighting the utility of native T1 mapping in detecting lipid metabolism abnormalities in IUGR rats.

This study advances the understanding of how IUGR predisposes individuals to adult obesity and liver pathology by linking altered lipin-1 and lipin-2 expression to disrupted hepatic lipid metabolism. The findings highlight how these molecular changes contribute to hepatic steatosis, fibrosis, and metabolic dysfunction, conditions commonly observed in IUGR individuals. By correlating lipin-1 and lipin-2 expression with hepatic T1 values, this study provides a novel, non-invasive method to detect and monitor early liver abnormalities, bridging molecular biology and clinical practice. This research emphasizes the role of lipid dysregulation in metabolic syndrome and highlights potential targets for early intervention to mitigate long-term risks associated with IUGR. This study presents novel findings by demonstrating a specific correlation between lipin-1 and lipin-2 expression levels and hepatic T1 values, offering new insights into liver health assessment. Elevated lipin-1 and lipin-2 expression was linked to increased hepatic lipid accumulation, fibrosis, and inflammation, leading to elevated T1 values due to increased water content in the extracellular matrix. This is the first study to establish a direct relationship between lipin-1 and lipin-2-mediated lipid dysregulation and measurable changes in hepatic T1 values, highlighting T1 mapping as a non-invasive biomarker for assessing liver abnormalities in IUGR individuals.

However, there are some limitations to this study. First, the rats self-regulated their food intake, and no significant differences in consumption were observed between IUGR and control rats. Second, we used a clinical wide-bore MRI scanner instead of a dedicated small animal scanner due to availability constraints at our institute. Third, the rats were allowed to breathe freely during imaging, which may have contributed to erroneous MR signals from other tissues being misattributed to the liver. Fourth, the high standard deviation (SD) values, exceeding 30% for lipin-1 and lipin-2 protein and mRNA gene expression in the control group, suggest significant variability in the data. This variability could be due to biological differences, experimental conditions, or measurement techniques. To reduce variability in future experiments, we plan to increase the sample size and explore normalization strategies. Lastly, while we investigated the correlation between lipin expression and hepatic lipid metabolism mechanisms, additional biochemical analyses, such as lipid profiling in plasma and adipose tissue and measurements of antioxidant and inflammatory biomarkers, were not included in the current study. Future research could address these limitations by implementing controlled feeding protocols, using dedicated small animal MRI scanners for higher resolution imaging, incorporating respiratory gating techniques to improve signal specificity in liver T1 mapping, and expanding the scope of biochemical analyses. These improvements would enhance the reliability of native T1 mapping in preclinical and clinical research, advancing our understanding of metabolic adaptations in IUGR and related conditions.

To gain a more comprehensive understanding of the impact of IUGR on metabolic health, future research could explore additional molecular markers, such as those involved in inflammation, oxidative stress, and insulin resistance, to identify broader metabolic disruptions. Examining key metabolic genes, including those related to glucose metabolism and lipid storage, could offer insight into the molecular underpinnings of IUGR. Additionally, advanced imaging techniques like magnetic resonance spectroscopy (MRS) or positron emission tomography (PET) could be used to assess tissue-specific metabolic changes *in vivo*, allowing for real-time monitoring of liver fat content, muscle function, and other metabolic parameters. Combining these molecular and imaging approaches would provide a more holistic view of the long-term effects of IUGR on metabolic health.

Future research could focus on conducting larger cohort studies to validate findings and ensure robustness across different populations, including exploring the effects in female rats to identify potential sex-specific mechanisms. Mechanistic studies should delve deeper into the molecular pathways influenced by puerarin, particularly in lipid metabolism and liver fat accumulation, using transcriptomic and proteomic approaches. Expanding research to other tissues, such as adipose, muscle, and brain tissues, could reveal systemic metabolic effects. Comparative studies in different animal models, including larger mammals, would help assess the generalizability of findings, while pilot human trials could determine the clinical relevance of puerarin, particularly in metabolic disorders like NAFLD and obesity.

## Figures and Tables

**Table 1:** Comparison of T1 mapping and Lipin-1 and Lipin-2 mRNA and protein expression in liver and visceral adipose tissues of IUGR and control rats.

-	3-Week		8-Week		12-Week	
Variables	Control	IUGR	Control	IUGR	Control	IUGR
Lipin-1 protein	0.3366±0.1737	0.6599±0.0572*	0.4912±0.0809	0.7302±0.1430*	0.5230±0.1949	0.8791±0.1234*
Lipin-1 mRNA	1.0125±0.1730	1.2770±0.0820*	1.2091±0.2812	1.4350±0.1612*	1.2260±0.1847	1.5273±0.1397*
Lipin-2 protein	0.5665±0.3480	0.9305±0.3266*	0.9651±0.3194	1.2305±0.2197*	0.9471±0.0866	1.4686 0.3926*
Lipin-2 mRNA	1.1975±0.1312	1.4007±0.2365*	1.1810±0.0712	1.5771±0.2580*	1.2406±0.0541	1.820±0.1446*
T1 values (ms)	743.25±72.59	823.60±82.1*	768.90±90.40	933.70±31.33*	857.72±106.61	963.70±56.33*
BMI (kg/m^2^)	5.48±0.61*	4.47±0.45	6.565±0.578*	7.552±0.569	7.615±0.641*	8.679±0.404
BL (cm)	10.42±0.32*	9.53±0.42	18.56±0.65	18.45±0.599	21.95±0.76*	23.10±1.59
WC (cm)	9.4±0.23	9.5±0.38	16.400±0.718*	17.19±0.523	20.10±0.567	20.80±0.632
Weight (g)	59.2±4.3*	47.3±6.3	219.79±25.29*	247.04±22.77	347.62±42.92*	424.43±34.08

Data were presented as mean ± SD (*n* = 10), *P < 0.05, includes *P < 0.001 and 0.0001, IUGR *vs.* control.

## Data Availability

The data and supportive information are available within the article. (Data private access link: https://www.scidb.cn/en/s/AzqUZb; data anonymous private link;https://www.scidb.cn/en/anonymous/QxpxVVpi).

## LIST OF ABBREVIATIONS

IUGR: Intrauterine growth restriction
MR: Magnetic resonance
SD: Sprague-Dawley
BMI: Body Mass Index
WB: Western Blot
